# Investigating the Impact of Possession-Way of a Smartphone on Action Recognition †

**DOI:** 10.3390/s16060812

**Published:** 2016-06-02

**Authors:** Zae Myung Kim, Young-Seob Jeong, Hyung Rai Oh, Kyo-Joong Oh, Chae-Gyun Lim, Youssef Iraqi, Ho-Jin Choi

**Affiliations:** 1 School of Computing, KAIST, Daejeon 34141, Korea; zaemyung@kaist.ac.kr (Z.M.K.); aomaru@kaist.ac.kr (K.-J.O.); rayote@kaist.ac.kr (C.-G.L.); 2 Naver Labs, Seongnam 13561, Korea; pinode.waider@navercorp.com; 3 Samsung Electronics, Seoul 06765, Korea; hyungrai.oh@samsung.com; 4 Electrical and Computer Engineering Department, Khalifa University, Abu Dhabi 127788, UAE; Youssef.Iraqi@kustar.ac.ae

**Keywords:** action recognition, possession-way recognition, artificial neural networks

## Abstract

For the past few decades, action recognition has been attracting many researchers due to its wide use in a variety of applications. Especially with the increasing number of smartphone users, many studies have been conducted using sensors within a smartphone. However, a lot of these studies assume that the users carry the device in specific ways such as by hand, in a pocket, in a bag, *etc*. This paper investigates the impact of providing an action recognition system with the information of the possession-way of a smartphone, and *vice versa*. The experimental dataset consists of five possession-ways (hand, backpack, upper-pocket, lower-pocket, and shoulder-bag) and two actions (walking and running) gathered by seven users separately. Various machine learning models including recurrent neural network architectures are employed to explore the relationship between the action recognition and the possession-way recognition. The experimental results show that the assumption of possession-ways of smartphones do affect the performance of action recognition, and *vice versa*. The results also reveal that a good performance is achieved when both actions and possession-ways are recognized simultaneously.

## 1. Introduction

With the advances in technology, a lot of research efforts have been put into developing autonomous systems for convenient human lifestyles [1,2,3]. Developing such systems involves effective modeling of human behavior as the systems are required to conduct many things for humans. Action recognition is one of these techniques, and has been utilized in diverse applications such as health-care monitoring systems [4,5] and surveillance systems [6,7].

Although there are many existing studies on developing action recognition systems using wearable sensors, most of them are not so practical as the wearers may find it troublesome to wear and carry the sensors around with them. In order to meet practical needs, along with the growing number of smartphone users, many studies in action recognition have been conducted using smartphones, which are equipped with various types of sensors such as accelerometers, gyroscopes, light sensors, *etc*. Since many users carry their phones closely with them all the time, utilizing the devices for action recognition seems more practical than wearing extra sensors.

However, many existing works in action recognition using smartphones are somewhat limited in the sense that they commonly assume that the users possess or carry the devices in certain ways, for example, by hand, in a pocket, in a bag, *etc*. This assumption is difficult to hold in the real world as different users carry their phones in different ways. Furthermore, even the same user may carry the phone differently in various situations. For example, a user may prefer to use the phone while walking, in which case he/she holds the phone with a hand; however, while running, the user may prefer to put the device in a pocket or a bag.

In this paper, we extend our previous work [8] to investigate the relationship between the action recognition and possession-way recognition using smartphones, adopting a larger dataset than the previous work and additionally utilizing state-of-the-art algorithms that are designed to learn the temporal dependencies among the sensed data. Seven users were recruited to gather the sensor data separately; they are asked to perform two actions (*walking* and *running*) while carrying their phones in five different ways (by *hand*, in a *backpack*, in an *upper-pocket*, in a *lower-pocket*, and in a *shoulder-bag*).

Experiments with the larger dataset confirm our previous findings that simultaneously recognizing both actions and possession-way improves the overall performance. In addition, we closely investigate how the performance varies in accordance with the length and the number of time intervals (windows) that features are computed for, as well as with various machine learning algorithms.

The remainder of the paper is organized as follows. Section 2 outlines the background for this study, while Section 3 describes the proposed approaches in detail. We illustrate the experiments and present the results in Section 4, and discuss the experimental findings in Section 5. Finally, the paper is concluded in Section 6 with some directions for future works.

## 2. Background

Over the past few decades, a lot of studies have been conducted in the field of action recognition. These works can be arranged into three categories according to the form of data they employ: (1) images [9,10,11]; (2) videos [12,13,14]; and (3) a variety of sensor data [15,16,17,18,19,20]. This paper comes under the third category: action recognition using sensor inputs. Typically, the input streams utilized in such works are obtained from wearable sensors in the form of wrist-type, pad-type, or necklace-type sensors. Such studies that make use of these wearable sensors have demonstrated high accuracy in action recognition.

However, one major drawback of using these wearable sensors in everyday application is that the users often feel uncomfortable wearing them. In addition, having to remember to wear the devices can be troublesome as well. This issue is especially critical in the health-care domain; an effective sensor device does not only refer to a device with technical and clinical advantage, but also the one that the end-consumers find acceptable to wear [21]. In other words, designing a good wearable sensor requires a careful assessment of user wearability (usability), which is another important topic but quite different to the topic of improving the technical performance of sensor applications.

In this work, we instead focus on an alternative device that most people do not live without—the smartphone. In South Korea, it is estimated that 83.0% of Koreans own a smartphone, and on average, spend 3 h and 39 min a day purely on using smartphones [22]. Smartphones have become so integral to our life that most people find it natural to carry them around all the time. Furthermore, as they are equipped with various types of sensors (e.g., accelerometer, gyroscopes, proximity sensor, *etc*.), exploring diverse combinations of sensor inputs is possible.

As a matter of fact, much research has been done on action recognition using the sensors of smartphones over the past few years. For example, Dai *et al*. [23] proposed a pervasive fall detection system on mobile phones called *PerFallD*, utilizing the accelerometer sensor and magnetic field sensor of smartphones, and an additional magnetic field accessory.

More recently, He *et al*. [24] developed another fall detection system solely on smartphones, which notifies the caregivers of the fall accidents through Multimedia Messaging Service (MMS) containing a map of suspected location and time. Using the built-in tri-accelerometer, their system classifies five body motions—vertical activity, lying, sitting or standing, horizontal activity, and fall. However, it assumes that the smartphone is mounted on the user’s waist.

Song *et al*. [25] analyzed users’ daily behaviors in terms of movements (e.g., sit down, run), actions (e.g., phone call, read mail), and situations over time (e.g., home, car, subway). An HMM (Hidden Markov Model)-like model is trained from users’ activities over a series of days, and utilized to extract behavior patterns by time-movement correlation, time-action correlation, *etc*.

The aforementioned studies have shown the possibility of action recognition using the smartphones. However, they commonly assume that the possession-way of a smartphone is fixed, for example, by hand, in a pocket, in a bag, *etc*. In reality, users carry their phones differently in various situations; thus, it is necessary to develop action recognition systems that are invariant to the possession-ways. Inspired by such a viewpoint, this paper investigates the following questions:

1. “How much impact does the information of possession-way of smartphones have on the action recognition?”
2. “Likewise, how much does the recognized action influence the recognition of the possession-way of a smartphone?”
3. “If each task does indeed influence the other, which one should be carried out first (performance-wise)?”

To the best of our knowledge, our published conference paper [8] was the first study to delve into these questions. We hope that our findings can help the other related tasks that involve action recognition using smartphones, as the results provide information in dealing with the possession-ways of the device.

## 3. Proposed Method

### 3.1. Overall Approach

The purpose of this study is to investigate the relationship between the action recognition and the possession-way recognition using the smartphones. To do so, we propose three experimental approaches, and compare the results:

1. Conducting the possession-way recognition followed by the action recognition.
2. Conducting the action recognition followed by the possession-way recognition.
3. Conducting both of the recognition tasks simultaneously.

**Figure 1:**
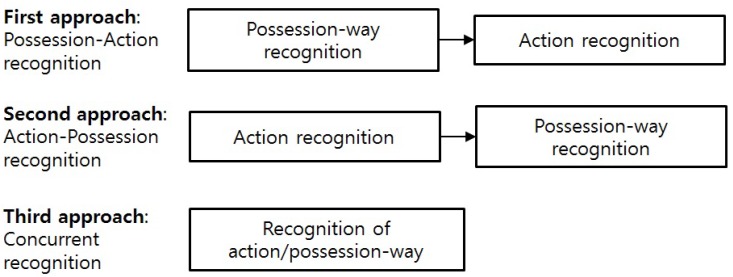
Three proposed approaches.

Let us assume that there are *A* actions and *P* possession-ways. Given an unseen piece of data *X*, these approaches aim to find which action is performed (action recognition), and how the smartphone is carried by the user (possession-way recognition). Figure 1 summarizes these approaches.

The first approach, *possession-action recognition*, consists of two steps. In the first step, it recognizes the possession-way of the unseen data *X* without considering the action. This step is simply a classification task over *P* classes. The second step is to recognize the action given the recognized possession-way, so it can be seen as a classification task over *A* classes. Note that if the recognized possession-way in the first step is incorrect, then it may deteriorate the performance of the action recognition in the second step.

The second approach, *action-possession recognition*, also has two steps. The first step refers to the classification over *A* classes, and the second step, the classification over *P* classes. If one wishes to develop only an action recognition system, then the second step is not necessary as the action is recognized in the first step.

The third approach, *concurrent recognition*, involves classifying the action and the possession-way simultaneously. Therefore, it is a one-step classification over A×P classes.

One may argue that the action recognition using smartphones is inherently a challenging task as it involves dealing with the direction or the angle of the smartphone carried by the user. Fortunately, advances in hardware have equipped the phones with gyroscope sensors which compute the angle of the device, making it easy to access the angular data.

### 3.2. Feature Definitions

Let us denote the number of sensor dimensions to be *S*. The exact number may vary with different models of smartphones, as well as the versions of Android API (Application Programming Interface); we describe the details of the sensor types in Section 4.3.

Given a window size of *W* seconds, we compute the *mean*, *minimum*, *maximum*, and *variance* values for each window. Therefore, for each window, we have 4×S distinct features for *S* sensor dimensions. The feature engineering is kept simple as the main focus is on the verification of the relationship between the action recognition and possession-way recognition using the smartphones.

### 3.3. Classification Algorithms

We employ five classification algorithms: naive Bayes (NB), random forests (RF), support vector machine (SVM), deep neural networks (DNN), and recurrent neural networks (RNN) to compare the performances of the three approaches. Note that, in our previous work, we utilized three classification algorithms: naive Bayes, decision trees (DT), and artificial neural networks with one hidden layer. However, we empirically found that the extended dataset of this study required more powerful machine learning algorithms to effectively learn the greater variances in the data gathered by the seven users. Therefore, we added RF instead of DT, along with SVM, DNN, and RNN models. The hyper-parameter settings of these algorithms, which were determined by grid searching, are as follows:

- NB: Gaussian.
- RF: 100 decision trees, Gini impurity.
- SVM: Liblinear, l2 penalty, hinge loss, tolerance of 0.0001.

**Figure 2:**
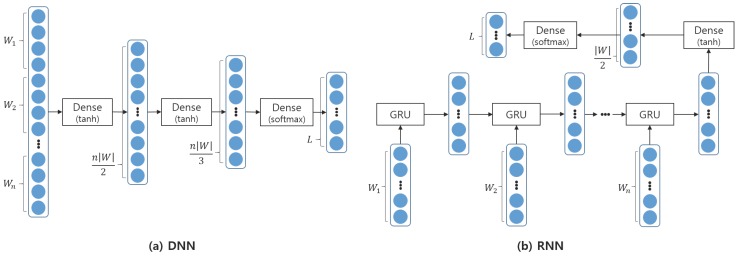
The proposed structures of (**a**) DNN and (**b**) RNN.

The structure of DNN and RNN models are depicted in Figure 2. The input layer of the DNN model takes *n* feature windows, each consisting of 4×S distinct features as described in Section 3.2. Note that an experimental analysis on this parameter *n* is conducted in Section 4.4, Section 4.5 and Section 4.6, where we observe the changes in the performance of each approach when *n* is varied.

The DNN model has two fully connected hidden layers with a hyperbolic tangent (tanh) as the activation function. The dimensions of the first and the second hidden layers are $\frac{n\left|W\right|}{2}$ and $\frac{n\left|W\right|}{3}$, respectively. The softmax output layer classifies the instances as one of *L* class labels, where *L* can be *A*, *P* or A×P depending on the classification tasks.

The RNN model consists of a gated recurrent unit (GRU) [26] layer and two fully-connected layers. A GRU works in a similar fashion to its older cousin, long short-term memory (LSTM) units [27], in the sense that it adaptively *updates* or *resets* its memory content via gating mechanism. Nevertheless, the GRU has a slightly simpler structure than LSTM, often reducing the overall time in training. For this reason, we chose to employ GRU, and it indeed converged faster than LSTM without sacrificing the performance.

The GRU layer is structured in a many-to-one fashion, meaning that only the last hidden state is passed on to the next layer. Similar to the input dimension of the DNN model, *n* feature windows are fed in as *n* time steps. The fully connected hidden layer outputs a vector of $\frac{n\left|W\right|}{2}$ dimensions, which is, in turn, classified into one of *A*, *P* or A×P class labels.

## 4. Experiments

### 4.1. Dataset Construction

**Figure 3:**
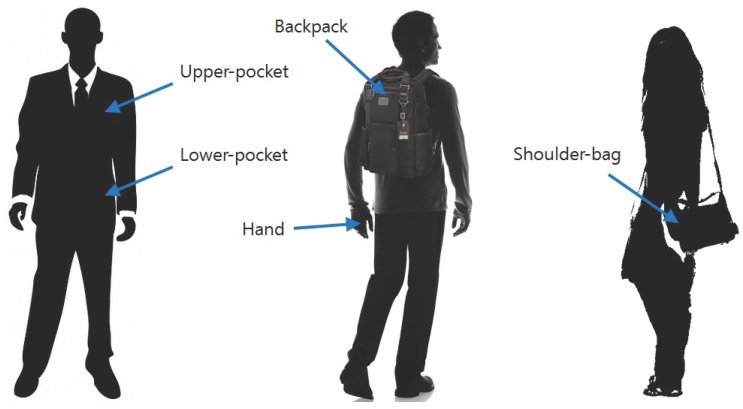
The five possession-ways of a smartphone targeted in the experiments.

As there are no publicly available dataset for this study, we have gathered a dataset by implementing an Android application that continuously logs the sensor values of a smartphone. We target two actions, *walk* and *run* and five possession-ways of a smartphone: *hand*, *backpack*, *upper-pocket*, *lower-pocket*, and *shoulder-bag* (Figure 3).

In our previous study [8], the dataset is collected for a single user only, using a Samsung Galaxy Nexus smartphone, (Samsung Electronics, Suwon, South Korea); in this study, the new dataset is gathered by seven users, using various Android-based smartphones. We note that the seven participants were volunteers from the department of computer science. The participants had a wide range of physical attributes in terms of gender, age (23 to 33 years old), height (167 cm to 182 cm), and weight (45 kg to 101 kg), which we believed to be relevant factors in our tasks as these attributes may influence the frequency and amplitude of the gathered sensor data.

Identical to the previous study, each user performed each action for 10–11 min. However, in this study, each sensor value is recorded at the sampling rate of 40 Hz instead of 10 Hz.

The statistics of the raw dataset are described in Table 1 where the values represent the number of gathered samples; as each action is performed for 10–11 min by seven users, the number of gathered samples is approximately 40×60×10×7 for each action-possession combination.

### 4.2. Preprocessing the Dataset

Prior to generating features, the raw dataset has undergone three preprocessing steps. Firstly, the sensor values from the eight sensors are interpolated with 100 millisecond intervals. Although the sensors are programmed to measure a value every 40 Hz, in reality, all measurements are not perfectly synchronized. Therefore, we take the initial time stamp of the very last sensor that begins to measure as our starting time stamp for all sensors. Similarly, the earliest final time stamp of a sensor is taken as the finishing time stamp for all sensors as well. Given the time intervals, linear interpolation is conducted for all sensors that measure continuous real values. The three sensors, *light*, *proximity*, and *pressure*, provide two discrete values, either 0 or a fixed integer smaller than 10. For the three sensors, the nearest neighbor approach is taken to fill the missing values in the time range.

Secondly, the raw values are normalized by min-max normalization where the values ($v_{raw}$) are linearly transformed to fit a given range, $\left[r_{1},r_{2}\right]$:

(1) $$v_{scaled}=\frac{\left(v_{raw}-min\left(v_{raw}\right)\right)}{\left(max\left(v_{raw}\right)-min\left(v_{raw}\right)\right)}\times \left(r_{1}-r_{2}\right)+r_{2}$$

The minimum and maximum values of each sensor are obtained by consulting the relevant materials on the Android API documentations.

Lastly, we disregard the first and the last 15 s of the data, as users generally took such time to begin or end the data logging. At the end of preprocessing, the normalized values from the eight sensors (19 distinct values in total) are lined up on one coherent time line with 100 millisecond interval.

### 4.3. Feature Generation

In Section 3.2, we explained that the four features, (*mean*, *minimum*, *maximum*, and *variance*), are computed using the *S* sensor dimensions for each window *W*. We employ eight sensors that are equipped in a typical smartphone: *light*, *proximity*, *pressure*, *gravity*, *accelerometer*, *linear accelerometer*, *gyroscope*, and *rotation* sensors (we mention that two sensors, *orientation* and *magnetic*, are no longer utilized in this study due to API mismatch between the Android application and the different phones). The *light*, *proximity*, and *pressure* sensors generate a one dimensional real value, while the *rotation* sensor generates four dimensional real values. The other remaining sensors produce three dimensional real values. In total, we have S=19 sensor dimensions, and 76 features (4×19) obtained based on the feature definition.

We experiment with varying the *length of window* $\left|W\right|$ for which the 76 features are computed, and the window sizes are 0.3, 0.5, 1, 3, 5, and 7 s. For example, when $\left|W\right|=0.3$, the features are computed using the three rows of the data as each row is 100 milliseconds apart. The statistics of the generated feature samples are summarized in Table 2, where the values represent the number of samples after the feature generation. The number of generated feature samples decreases as the length of the window increases.

In addition to varying the length of the time window, we experiment with varying the *number of windows* while fixing the length of the window as 1 s ($\left|W\right|=1$). We clarify that the number of windows refers to how many consecutive sets of generated features are taken as the input, while the length of the window represents how many seconds of the raw data are used to generate one set of features. Evidently, varying the number of windows will produce input vectors with different lengths. For each classification task, experiments are performed by setting the number of windows *n* as 1, 3, 5, and 7.

Besides the two experiments, we also look at the performance of each model when applied to a new user who has not been considered by the model in the training process, *i.e.*, one-user-out cross validation (CV). The data gathered by six users are utilized in the training process, while the data from the remaining one user is used as the testing data. Since there are seven users in total, we have seven rounds of validation, and the results are averaged for each model. Note that for the one-user-out CV, we set the length of window $\left|W\right|$ to be 1 and the number of window *n* to be 3.

**Figure 4:**
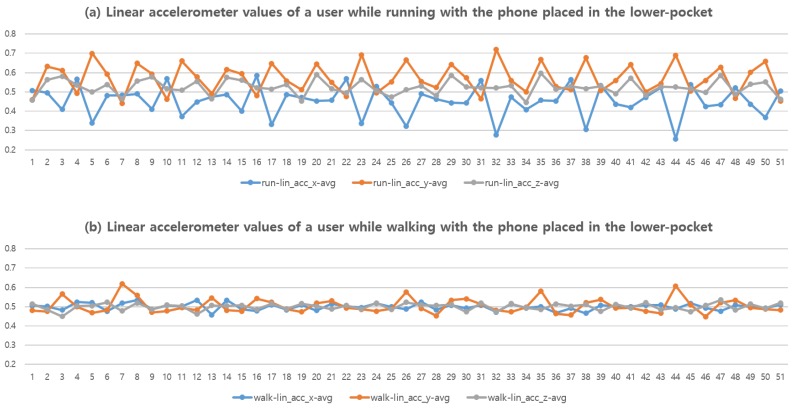
Linear accelerometer values of a user while (**a**) running and (**b**) walking with the phone placed in the lower-pocket.

Figure 4 illustrates two sample graphs of the linearized tri-accelerometer values of a user while (a) *running* and (b) *walking* with the phone placed in a lower-pocket. The graphs are plotted with 50 samples, where each sample represents the mean value of a 0.5 s interval. We can observe that the amplitude of *running* is greater than that of *walking*.

While differentiating the two actions from a single user seems to be apparent, the task gets harder when more users are involved. For example, some of the users were fast-walkers, and their patterns were closely akin to the running patterns of slow runners.

### 4.4. Possession-Action Recognition

The first approach, possession-action recognition, consists of two steps: (1) possession-way recognition, and (2) action recognition. The five classification algorithms described in Section 3.3 are employed to compare the performances under fivefold (Table 3 and Table 4) and one-user-out (Table 5) CV after shuffling the dataset. Table 3 presents the experimental results for the different lengths of the windows, while Table 4 shows the results for the different number of windows. The results for the one-user-out CV are presented in Table 5. *Step 1* and *Step 2* in both tables indicate the accuracies of possession-way recognition and action recognition, respectively. For example, *Step 2 (hand)* shows the accuracies of action recognition when the possession-way is by *hand*.

We specify that the following abbreviations are used in the subsequent tables: support vector machine (SVM), random forests (RF), naive Bayes (NB), deep neural network (DNN), and recurrent neural network (RNN).

Notably, under the fivefold CV, the random forests (RF) model works consistently well, outperforming the two deep learning approaches on almost all settings. However, as we have not sufficiently explored the many possible layouts and hyper-parameter settings of the deep learning models, we cannot decide the superiority of one model over another in terms of accuracy. Moreover, it is often acknowledged in the literature [28,29] that a machine learning model typically requires training data for at least 10 times its degree of freedom. As the deep learning models consisted of a greater number of weight parameters than the other machine learning models, a lot more data samples would have been necessary for effective learning.

Nevertheless, it is important to note that the RF model only took a few seconds to train, while the deep learning models took considerably longer time (from a dozen minutes to hours depending on the number of inputs).

As depicted in both tables, the accuracies of *Step 1* are less than that of *Step 2*. This implies that the action recognition task (*Step 2*) becomes easier when the possession-way is given or assumed, as many existing studies have done so. The overall performance of action recognition is calculated by multiplying each (possession-way) accuracy of *Step 1* by the corresponding accuracy of *Step 2*, and computing the mean of the multiplied accuracies:

(2) $$accuracy_{overall}=\frac{\sum_{i\in P}Step_{1}\left(i\right)\times Step_{2}\left(i\right)}{5}$$

In the case of RF models, we can roughly see that the overall accuracy is 66 to 68.

Table 3 shows the accuracies of the five algorithms at varying lengths of the window. Each classification algorithm exhibits a slightly different pattern; however, in general, the accuracies are increasing as the window length reaches 3 s, and slightly deteriorate afterwards. The exception is RF models where the accuracies are consistently high at 82, reaching its best at $\left|W\right|=0.3$.

Table 4 shows the accuracies of the five algorithms for the different number of windows. Again, each classifier behaves differently with the increasing number of windows. For example, the accuracy of possession-way recognition (*Step 1*) of SVM decreases as *n* increases, in contrast to that of NB. Overall, the best performance is achieved by RF when n=1.

As shown in Table 5, the results of the one-user-out CV show quite a different trend. Firstly, the overall performance of the top three classification models, RF, RNN, and DNN, has dropped significantly due to the sharp decrease in the performance for *Step 1*, possession-way recognition. On the contrary, the performance for *Step 2*, action recognition, has actually increased by a small amount compared to the results obtained from the fivefold CV. The results show that the task of possession-way recognition for an unseen user is not a trivial one.

Currently, the process of our feature engineering is kept simple (Section 3.2) as the aim of the study is to explore the relationship between the three approaches. Improving the accuracy of the recognition tasks would require more thoughtful feature definitions. For example, as each participant carried his/her phone in an arbitrary orientation, it is possible that the some specific orientations of the phones, rather than their more general representation, could have been reflected on the models’ learning process. Therefore, a rotation-invariant feature [30] would be a good solution here.

It is also worth mentioning that the overall performance by the models on this dataset is lower than the performance on the previous dataset [8]. This is because the previous dataset only consisted of data from a single user, while this dataset is contributed by seven users. Therefore, the action recognition models learned from this dataset is more general than the models learned in the previous work.

### 4.5. Action–Possession Recognition

The second approach, action-possession recognition, also consists of two steps: (1) action recognition, and (2) possession-way recognition. Similar to the previous subsection, the five classification algorithms are employed and evaluated under both fivefold and one-user-out CV. The results are summarized in Table 6, Table 7 and Table 8.

*Step 1* in the tables represents the accuracies of action recognition, while *Step 2* shows the accuracies of possession-way recognition when the action is known. For instance, *Step 2 (walk)* shows the accuracies of possession-way recognition when the user is *walking*.

As shown in Table 6, the performance of action recognition (*Step 1*) generally gets better as the window length increases. Again, the RF is marked as an exception as its performance stays quite consistent throughout the experiments under fivefold CV.

Similar to the results of possession-action recognition (Section 4.4), the overall accuracies of one-user-out CV are lower than the ones obtained from fivefold CV, despite the increases in accuracy for *Step 1*, action recognition.

Under both evaluation criteria, most classifiers (with the exception of RF in fivefold CV) found it easier to recognize the possession-way of a smartphone when a *running* action is assumed. One possible explanation is that the influence from the surroundings of the smartphone is maximized when the user is running rather than walking, hence producing sensor values with richer information.

Similar to the possession-action recognition, the best performance is achieved by RF when the length of windows $\left|W\right|=0.5$ and the number of windows n=1.

Another important point to note is that the accuracies of *Step 1 (action recognition)* are generally lower than that of *Step 2 (action recognition)* in the possession-action recognition shown in Section 4.4. This is because *Step 1* of action-possession recognition involves directly classifying the actions regardless of the various possession-ways in which the actions are blended.

### 4.6. Concurrent Recognition

The third approach, concurrent recognition, aims to classify both actions and possession-ways of a smartphone simultaneously. The five classification algorithms are used again, and the results are described in Table 9, Table 10 and Table 11. In all tables, *Conc.*, *P-A*, and *A-P* refer to the overall accuracies of the concurrent, possession-action, and action-possession recognitions, respectively.

As the concurrent approach classifies A×P classes, the accuracies of most classifiers are lower than that of individual steps in the previous approaches, where *A* or *P* classes are recognized separately. Therefore, for a fair comparison, the mean values of the combined accuracies of the possession-action and action-possession recognitions are presented in the tables. Comparing the overall performance, the concurrent approach performs better than the rest of the two approaches even under the one-user-out CV (Table 11).

In general, the results show a similar trend with the previous two approaches: the performance increases with the lengths of windows, and decreases with the number of windows. The RF produces consistently high performance while the other algorithms are hindered by the increased number of class labels.

Similar to the previous two approaches, the extended dataset of this study results in lower performance than the single-user dataset from the previous study [8]. We also need to point out that the results of concurrent recognition in this study are slightly different to what we found in the previous study using the single-user dataset. In the previous study, the accuracy of concurrent recognition was as good as, or just slightly lower than, the accuracies of the separate tasks (*Step 1* and *Step 2*) in the two approaches. In this study, however, the concurrent recognition does indeed seem to be a harder task than the two tasks.

We believe that this was due to the fact that the single-user dataset had made all three of the tasks very easy. In the single-user dataset, the data samples for each action or possession are coherent to each other as they are from one user. In contrast, the samples gathered by the seven users are not necessarily coherent, as different users have their own style of walking, running, holding the phone, *etc*. Such difference in the datasets is illustrated clearly by the results of the one-user-out CV where the learned models had difficulty in recognizing the possession-ways of the smartphone for an unseen user.

## 5. Discussion

From the experiments, we have explored how the performance of a classifier varies according to the length and the number of windows for each classification task. Although the detailed patterns are a little different among classifiers, a few remarks about the general trend can be made:

- The length of windows ($\left|W\right|$) seems to be a more important factor in improving the performance than the number of windows (*n*). In other words, for the tasks of recognizing individual action and possession-way of a smartphone, the independent features computed from each time interval play a bigger role than a series of *n* features that represent the sequential patterns.
- In general, the performance of action recognition increases as the classifiers observe data for longer period of time, *i.e.*, greater *n* and $\left|W\right|$. In contrast, the performance of possession-way recognition is not so much affected, perhaps because the differences in patterns of possession-ways are not very significant to each other. For example, the sensor patterns obtained from placing the smartphone in a *shoulder-bag* may actually be similar to the pattern gathered by placing the device in a *backpack*; and increasing the length and number of windows would not affect the classification performance significantly.
- The concurrent approach seems to work best when *n* and $\left|W\right|$ are around 3 to 5.
- However, the RF remains an exception to these trends. While consistently producing the best results under the fivefold CV, it particularly works well when $\left|W\right|$ is 0.3 to 0.5. This is probably due to the inner workings of the RF; similar to tree bagging, the RF repeatedly selects a random sample from the training set, and fits trees to these samples. When $\left|W\right|$ is small, many data samples are available for the random sampling. We suspect that sampling from this larger pool results in the increased overall performance of the RF.
- The RNN performs the second best in the tasks under the fivefold CV. It is interesting to see that when $\left|W\right|$ or *n* are 1, its performance is as high as the model learned with longer data samples, illustrating the RNN’s capability to learn temporal patterns even with the shorter inputs.
- However, under the one-user-out CV, the performance of these top three models have decreased significantly for all three tasks. We suspect that, as these models tend to have a greater number of weight parameters to adjust, they had been overfitted to the training data. A greater number of training samples along with more careful feature definitions would be required to achieve a more stable performance.
- Nevertheless, under both one-user-out and fivefold CV, the concurrent approach produces better results than the other two approaches—possession-action and action-possession recognition.

## 6. Conclusions

We investigated the relationship between the action recognition and possession-way recognition using smartphones. In order to further investigate our previous findings [8], we extended the previous dataset to encompass sensor data from seven users rather than a single one. We proposed the three approaches—possession-action recognition, action-possession recognition, and concurrent recognition—and experimentally verified that the assumption of possession-way of the smartphone does affect the performance of action recognition, and *vice versa*. We observed that the concurrent recognition, which classifies both action and possession-way simultaneously, produces good results compared to the overall accuracies of the two other approaches.

For future work, conducting the same experiment with additional actions such as *cycling* would be interesting. Moreover, a series of user actions (*i.e.*, a long term behavior) rather than a single one could be learned, possibly taking full advantage of the power of RNN architectures or conditional random fields. However, we would need to utilize more advanced methods in feature engineering to stabilize the performance of both action and possession-way recognition for unseen users.

## Figures and Tables

**Table 1:** Statistics of the raw dataset.

	Hand	Backpack	Upper-Pocket	Lower-Pocket	Shoulder-Bag
**Run**	147,283	143,511	162,167	178,689	167,862
**Walk**	184,256	185,572	157,273	177,308	172,180

**Table 2:** Statistics of the generated feature samples.

		Hand	Backpack	Upper-Pocket	Lower-Pocket	Shoulder-Bag
$\left|W\right|=0.3$	**Run**	14144	14546	13554	15070	14291
	**Walk**	17769	15637	15880	15126	15380
$\left|W\right|=0.5$	**Run**	8484	8728	8130	9039	8573
	**Walk**	10661	9381	9526	9074	9227
$\left|W\right|=1$	**Run**	4240	4361	4062	4518	4283
	**Walk**	5328	4689	4761	4534	4612
$\left|W\right|=3$	**Run**	1409	1451	1349	1502	1424
	**Walk**	1774	1561	1583	1508	1534
$\left|W\right|=5$	**Run**	845	868	808	900	852
	**Walk**	1061	933	950	903	920
$\left|W\right|=7$	**Run**	601	619	577	641	607
	**Walk**	756	666	676	644	655

**Table 3:** Accuracies of possession-action recognition for different lengths of windows.

	$\left|W\right|=0.3$					$\left|W\right|=0.5$					$\left|W\right|=1$				
	SVM	RF	NB	DNN	RNN	SVM	RF	NB	DNN	RNN	SVM	RF	NB	DNN	RNN
**Step 1**	61.06	**82.67**	46.12	76.64	76.02	62.75	**82.57**	47.61	78.14	77.28	63.76	**82.45**	49.20	78.39	78.33
**Step 2 (backpack)**	75.70	**82.97**	73.73	80.24	80.74	76.70	**82.92**	77.45	80.20	80.81	78.43	**82.70**	79.59	81.65	80.68
**Step 2 (upper-pocket)**	78.17	**82.97**	69.41	81.40	81.26	80.11	**82.99**	74.17	81.85	82.12	81.60	**82.94**	78.05	81.88	82.16
**Step 2 (lower-pocket)**	77.02	**82.48**	69.53	78.83	79.58	78.76	**82.60**	72.88	80.55	80.10	79.42	**82.69**	75.77	80.85	79.78
**Step 2 (shoulder-bag)**	74.17	**83.08**	71.91	82.19	82.31	76.59	**82.95**	76.58	82.26	81.71	79.55	**82.83**	80.72	81.97	82.04
**Step 2 (hand)**	81.28	**82.94**	77.12	81.90	82.29	81.74	**82.92**	79.71	81.88	82.29	82.38	**82.98**	81.16	82.66	82.54
**Overall**	47.18	**68.52**	33.36	62.01	61.76	49.43	**68.43**	36.26	63.57	62.91	51.18	**68.29**	38.90	61.12	63.79
	**$\left|W\right|=3$**					**$\left|W\right|=5$**					**$\left|W\right|=7$**				
	**SVM**	**RF**	**NB**	**DNN**	**RNN**	**SVM**	**RF**	**NB**	**DNN**	**RNN**	**SVM**	**RF**	**NB**	**DNN**	**RNN**
**Step 1**	64.51	**82.14**	51.40	79.24	77.93	64.98	**81.71**	50.57	78.50	76.79	65.68	**81.73**	51.19	77.65	76.49
**Step 2 (backpack)**	80.76	**82.23**	80.26	81.48	81.06	81.16	**81.85**	80.56	81.02	81.30	81.19	**82.30**	80.80	81.58	81.45
**Step 2 (upper-pocket)**	82.59	**82.94**	80.60	82.42	82.34	82.57	**82.76**	80.39	82.39	82.43	82.40	**82.80**	80.47	82.07	82.60
**Step 2 (lower-pocket)**	80.90	**82.48**	77.63	81.17	80.51	81.21	**82.36**	78.39	81.62	81.90	81.06	**82.68**	78.92	81.91	81.91
**Step 2 (shoulder-bag)**	82.04	**82.71**	82.01	81.87	81.33	82.25	**82.53**	81.88	82.02	81.55	82.34	**82.61**	81.95	82.21	82.08
**Step 2 (hand)**	82.78	**82.94**	81.45	82.73	82.23	82.72	82.85	81.32	**82.94**	**82.94**	82.78	82.90	81.43	82.90	**83.03**
**Overall**	52.78	**67.90**	41.32	64.92	63.51	53.27	**67.39**	40.71	64.37	62.99	53.83	**67.56**	41.32	63.78	62.89

**Table 4:** Accuracies of possession-action recognition for different number of windows.

	n = 1					n = 3				
	SVM	RF	NB	DNN	RNN	SVM	RF	NB	DNN	RNN
**Step 1**	63.76	**82.45**	49.20	78.39	78.33	59.44	**81.95**	50.57	79.38	78.32
**Step 2 (backpack)**	78.43	**82.70**	79.59	81.65	80.68	80.35	**82.31**	79.96	81.26	80.71
**Step 2 (upper-pocket)**	81.60	**82.94**	78.05	81.88	82.16	82.06	**82.77**	80.18	82.37	82.25
**Step 2 (lower-pocket)**	79.42	**82.69**	75.77	80.85	79.78	80.46	**82.31**	77.03	81.45	81.65
**Step 2 (shoulder-bag)**	79.55	**82.83**	80.72	81.97	82.04	79.50	**82.69**	81.78	82.15	81.42
**Step 2 (hand)**	82.38	**82.98**	81.16	82.66	82.54	82.68	**82.84**	81.55	82.78	82.71
**Overall**	51.18	**68.29**	38.90	64.12	63.79	48.15	**67.68**	40.51	65.09	64.03
	**n = 5**					**n = 7**				
	**SVM**	**RF**	**NB**	**DNN**	**RNN**	**SVM**	**RF**	**NB**	**DNN**	**RNN**
**Step 1**	57.53	**81.45**	51.22	75.78	76.72	56.46	**81.41**	51.58	74.65	75.70
**Step 2 (backpack)**	80.28	**82.27**	79.87	78.71	81.02	80.35	**82.43**	79.77	79.44	81.13
**Step 2 (upper-pocket)**	82.53	**82.81**	80.21	**82.81**	82.67	82.40	**82.93**	80.74	82.80	82.60
**Step 2 (lower-pocket)**	80.47	81.81	77.60	**81.95**	79.78	80.48	**82.04**	78.02	81.65	81.65
**Step 2 (shoulder-bag)**	79.29	**82.86**	81.78	79.62	81.31	78.46	**82.54**	81.62	78.19	81.42
**Step 2 (hand)**	**82.81**	82.72	81.32	82.77	82.59	**82.90**	82.78	81.13	82.66	83.03
**Overall**	46.64	**67.19**	41.06	61.51	62.51	45.69	**67.20**	41.40	60.43	62.05

**Table 5:** Accuracies of possession-action recognition under one-user-out cross validation.

	SVM	RF	NB	DNN	RNN
**Step 1**	49.58	51.48	**52.29**	51.00	48.66
**Step 2 (backpack)**	**88.22**	73.37	77.47	82.68	78.27
**Step 2 (upper-pocket)**	87.50	81.43	75.33	**90.47**	89.55
**Step 2 (lower-pocket)**	**86.77**	84.41	80.81	85.22	83.70
**Step 2 (shoulder-bag)**	91.83	81.43	**93.68**	73.22	75.12
**Step 2 (hand)**	94.75	92.49	94.14	**97.98**	92.38
**Overall**	**44.53**	42.54	44.07	43.82	40.78

**Table 6:** Accuracies of action-possession recognition for different lengths of windows.

	$\left|W\right|=0.3$					$\left|W\right|=0.5$					$\left|W\right|=1$				
	SVM	RF	NB	DNN	RNN	SVM	RF	NB	DNN	RNN	SVM	RF	NB	DNN	RNN
**Step 1**	72.42	**82.45**	66.72	79.19	79.12	74.70	**82.50**	70.54	80.10	79.48	77.46	**82.44**	73.71	80.16	80.02
**Step 2 (run)**	67.46	**82.59**	53.91	77.55	77.46	69.04	**82.60**	58.74	78.77	78.15	72.33	**82.56**	63.98	79.45	79.16
**Step 2 (walk)**	62.87	**82.99**	49.45	78.91	77.69	62.59	**82.88**	50.90	79.64	78.09	63.19	**82.75**	53.28	79.85	79.09
**Overall**	47.19	**68.26**	34.48	61.95	61.38	49.16	**68.26**	38.67	63.44	62.09	52.49	**68.14**	43.22	63.85	63.32
	**$\left|W\right|=3$**					**$\left|W\right|=5$**					**$\left|W\right|=7$**				
	**SVM**	**RF**	**NB**	**DNN**	**RNN**	**SVM**	**RF**	**NB**	**DNN**	**RNN**	**SVM**	**RF**	**NB**	**DNN**	**RNN**
**Step 1**	80.71	**82.29**	76.51	80.65	80.94	81.42	**82.39**	76.68	80.20	81.35	81.77	**82.43**	76.90	81.47	81.28
**Step 2 (run)**	75.71	**82.02**	67.90	79.99	79.58	76.37	**81.79**	69.02	79.82	79.08	76.82	**81.42**	68.58	79.45	79.15
**Step 2 (walk)**	63.09	**82.33**	54.88	79.71	78.00	61.90	**82.21**	55.26	78.14	77.07	61.18	**82.03**	55.52	77.27	77.57
**Overall**	56.01	**67.62**	46.97	64.40	63.77	56.29	**67.56**	47.65	63.34	63.51	56.42	**67.37**	47.72	63.84	63.69

**Table 7:** Accuracies of action-possession recognition for different number of windows.

	n = 1					n = 3				
	SVM	RF	NB	DNN	RNN	SVM	RF	NB	DNN	RNN
**Step 1**	77.46	**82.44**	73.71	80.16	80.02	78.94	**82.20**	76.04	79.91	79.92
**Step 2 (run)**	72.33	**82.56**	63.98	79.45	79.16	70.62	**81.83**	66.90	79.32	78.50
**Step 2 (walk)**	63.19	**82.75**	53.28	79.85	79.09	56.19	**82.31**	56.43	79.27	77.79
**Overall**	52.49	**68.14**	43.22	63.85	63.32	50.05	**67.46**	46.89	63.36	62.45
	**n = 5**					**n = 7**				
	**SVM**	**RF**	**NB**	**DNN**	**RNN**	**SVM**	**RF**	**NB**	**DNN**	**RNN**
**Step 1**	79.09	**82.11**	76.30	80.43	80.74	79.10	**81.77**	76.68	79.42	80.80
**Step 2 (run)**	67.77	**81.33**	65.61	79.22	79.46	65.85	**80.77**	64.52	77.68	79.92
**Step 2 (walk)**	51.88	**81.92**	57.45	77.58	76.13	49.26	**81.59**	57.70	75.91	75.10
**Overall**	47.32	**67.02**	46.95	63.06	62.81	45.53	**66.38**	46.86	60.99	62.63

**Table 8:** Accuracies of action-possession recognition under one-user-out cross validation.

	SVM	RF	NB	DNN	RNN
**Step 1**	**90.46**	88.05	89.67	86.31	88.60
**Step 2 (run)**	50.81	**59.78**	51.49	56.11	56.02
**Step 2 (walk)**	43.41	44.76	**47.56**	40.80	45.46
**Overall**	42.62	**46.02**	44.41	41.82	44.96

**Table 9:** Accuracies of concurrent, possession-action, and action-possession recognition for different lengths of windows.

	$\left|W\right|=0.3$					$\left|W\right|=0.5$					$\left|W\right|=1$				
	SVM	RF	NB	DNN	RNN	SVM	RF	NB	DNN	RNN	SVM	RF	NB	DNN	RNN
**Conc.**	60.07	**82.40**	43.32	75.38	74.46	61.65	**82.36**	48.37	76.55	76.59	64.64	**82.27**	54.14	78.00	78.08
**P-A**	47.18	**68.52**	33.36	62.01	61.76	49.43	**68.43**	36.26	63.57	62.91	51.18	**68.29**	38.90	64.12	63.79
**A-P**	47.19	**68.26**	34.48	61.95	61.38	49.16	**68.26**	38.67	63.44	62.09	52.49	**68.14**	43.22	63.85	63.32
	**$\left|W\right|=3$**					**$\left|W\right|=5$**					**$\left|W\right|=7$**				
	**SVM**	**RF**	**NB**	**DNN**	**RNN**	**SVM**	**RF**	**NB**	**DNN**	**RNN**	**SVM**	**RF**	**NB**	**DNN**	**RNN**
**Conc.**	67.85	**81.75**	58.52	78.25	77.79	67.92	**81.36**	59.37	78.16	77.42	67.90	**81.51**	58.55	77.84	76.53
**P-A**	52.78	**67.90**	41.32	64.92	63.51	53.27	**67.39**	40.71	64.37	62.99	53.83	**67.56**	41.32	63.78	62.89
**A-P**	56.01	**67.62**	46.97	64.40	63.77	56.29	**67.56**	47.65	63.34	63.51	56.42	**67.37**	47.72	63.84	63.69

**Table 10:** Accuracies of concurrent, possession-action, and action-possession recognition for different number of windows.

	n = 1					n = 3				
	SVM	RF	NB	DNN	RNN	SVM	RF	NB	DNN	RNN
**Conc.**	64.64	**82.27**	54.14	78.00	78.08	61.70	**81.43**	58.58	78.80	78.04
**P-A**	51.18	**68.29**	38.90	64.12	63.79	48.15	**67.68**	40.51	65.09	64.03
**A-P**	52.49	**68.14**	43.22	63.85	63.32	50.05	**67.46**	46.89	63.36	62.45
	**n = 5**					**n = 7**				
	**SVM**	**RF**	**NB**	**DNN**	**RNN**	**SVM**	**RF**	**NB**	**DNN**	**RNN**
**Conc.**	58.59	**81.27**	59.31	77.88	76.80	55.55	**80.85**	58.94	76.90	76.05
**P-A**	46.64	**67.19**	41.06	61.51	62.51	45.69	**67.20**	41.40	60.43	62.05
**A-P**	47.32	**67.02**	46.95	63.06	62.81	45.53	**66.38**	46.86	60.99	62.63

**Table 11:** Accuracies of concurrent, possession-action, and action-possession recognition under one-user-out cross validation.

	SVM	RF	NB	DNN	RNN
**Conc.**	44.73	**48.19**	44.82	44.88	47.48
**P-A**	**44.53**	42.54	44.07	43.82	40.78
**A-P**	42.62	**46.02**	44.41	41.82	44.96

## Abbreviations

The following abbreviations are used in this manuscript:

NB	Naive Bayes
DT	Decision Tree
RF	Random Forests
SVM	Support Vector Machine
ANN	Artificial Neural Network
DNN	Deep Neural Network
RNN	Recurrent Neural Network
LSTM	Long-Short Term Memory
GRU	Gated Recurrent Unit
